# Removal of the Inferior Maxilla

**Published:** 1865-01

**Authors:** 


Removal of the Inferior Maxilla.—The following cases of removal of the inferior maxilla, are remarkable for the rapidity of cure, and also the facility with which nature accomodates herself to circumstances.

Case 1. Corporal A. B., Co. I., Fourteenth Michigan Vet. Vol. Infantry, was struck by a musket ball on the chin, shattering the right portion of the inferior maxilla, and driving several pieces of bone and teeth through the cheek and into the soft palate. An incision was made from the angle of the jaw along the lower edge of the ramus to the symphysis, joined by one from the middle of the lower lip, and the necessary dissection made to isolate the bone. The chain saw was applied at the angle and at the symphisis and the bone removed. The facial artery was the only vessel tied. No chloroform was used, and in *twelve hours the patient was around* administering to the necessities of his wounded comrades, and at the end of a month was at his home, the wound perfectly healed and capable of using the ordinary fare.

Case 2. Sergeant G., Tenth Michigan Vet. Vol. Infantry on the 1st of September was shot through the inferior maxilla fracturing the bone and lacerating the soft parts. As in the above case the incision was made along the lower edge of the ramus from angle to angle, the bone divided and dissected from its muscular attachments and removed. The edges of the wound were brought together with hare-lip needles, and in six weeks his jaw was entirely healed. It was curious to observe the manner in which this man eat hard substances, such as meat and bread. He seemed *to hold* the morsel in the tongue, whilst with his upper teeth he soon reduced or rather "rasped" it down, the tissues became consolidated and will doubtless assume a fibro-cartilaginous character. No attempt at retraction of the tongue took place, nor have I yet seen a single instance, (though operating in some eight cases,) in which there was the slightest tendency to this accident so forcibly dwelt on by writers.

Case 3. Lt. A., Adjutant of Tenth Michigan Vet. Infantry, received a minnie ball through the lower edge of the ramus of the inferior maxilla, comminuting the bone and tearing the soft parts so dreadfully, that the tongue lay on the sternum, presenting all the appearance of a truly unpromising case. However the bones were removed and the ends sawn off at the angles; the edges of the wound were brought together and retained by the interrupted suture. At the end of three weeks all had united except at the lower extremity of the right angle where the loss of substance had formed a fistulous opening. I loosened the surrounding tissues, having made an incision through the opening, of about one and a half inches long, and bringing the edges that I had previously pared, together, retained them in position with pins and the twisted suture. At the end of six weeks he was enabled to return to his home, his face perfectly healed, and his general health not at all impaired. A fibrous tissue appeared to take the place of the bone, and afforded considerable resistance to the upper teeth to masticate food against, but as in the former case the tongue seemed to be the chief agent made use of by nature to compensate for the loss of the lower jaw.

The first idea that one gets on seeing the frightful wounds which occur about the face, is that of necessity, the larger portion of them must prove fatal. Such, however, is not the case as experience proves that death is the exception and recovery the rule.

